# Menstrual-Related Symptoms, Early Menarche, and Their Impact on Sport Participation in Adolescent Floorball Players: A Cross-Sectional Study in Sweden

**DOI:** 10.3390/sports14080358

**Published:** 2026-08-18

**Authors:** Eva Åkerman, Taru Tervo, Marie Klingberg-Allvin

**Affiliations:** 1 Department of Women’s and Children’s Health, Karolinska Institutet, SE-171 77 Solna, Sweden; marie.klingberg-allvin@ki.se; 2 Floorball Research and Development Centre, Umeå School of Sports Sciences, Umeå University, SE-901 87 Umeå, Sweden; taru.tervo@umu.se

**Keywords:** adolescent girl, menarche, menstrual health, floorball, menstrual management, sport experience

## Abstract

Purpose: This study aimed to examine the impact of menstrual symptoms on sport participation and management strategies among adolescent girls playing floorball in Sweden. Methods: A cross-sectional study was conducted among floorball players aged 12–18 years (*n* = 545). Logistic regression was used to assess factors associated with menstrual-related symptoms and their impact on participation and absence from training sessions or matches. Results: Menstrual pain (89.6%) and mood swings (81.9%) were the most commonly reported symptoms. Symptoms such as menstrual pain, heavy menstrual bleeding, headache, fatigue, dizziness, and concentration difficulties were more prevalent among participants with early menarche (≤11 years) than those with later menarche. Overall, about 62.2% perceived that their menstrual symptoms affected their training and matches. Early menarche (≤11 years) was associated with nearly three times higher odds of refraining from training sessions or matches. Despite substantial impact, healthcare-seeking and communication with coaches were limited. Conclusions: Given the high prevalence of menstrual symptoms and their impact on sport participation and experiences, menstrual health should be recognised as a core component of athlete well-being in youth sport. Efforts are needed to improve menstrual health awareness, early identification of girls with high symptom burden, and supportive sport environments to promote gender equality in youth sport.

## 1. Introduction

Menstrual health is defined as a state of complete physical, mental, and social well-being in relation to the menstrual cycle [1]. The menstrual cycle refers to the recurring physiological process from the first day of menstruation to the day preceding the next. Achieving menstrual health requires access to accurate and age-appropriate information; affordable menstrual products and adequate water, sanitation, and hygiene facilities; timely diagnosis and management of menstrual-related discomforts and disorders; a supportive social environment free from stigma and shame; and the ability to participate fully in education, work, and social life without discrimination. This study adopts this holistic conceptualization of menstrual health.

Menstrual health has historically been a taboo and under-researched topic in sports contexts [2]. Despite growing scholarly attention, research focusing on non-elite adolescent girls active in organised sports remains limited. Physical activity is associated with a wide range of physical and psychological health benefits among adolescents [3,4]. However, adolescent girls generally report lower levels of physical activity than boys [5], with a marked decline observed around puberty. This period also coincides with menarche, which typically occurs around a median age of 12–13 years [6,7]. Menstruation is associated with a range of physical and psychological symptoms, including dysmenorrhea (menstrual cramping), heavy menstrual bleeding (HMB), mood changes, headaches, fatigue, all of which are known to affect the daily lives of those who menstruate [8,9]. Dysmenorrhea is one of the most common menstrual complaints among adolescents [10], often accompanied by diarrhoea, back pain, headache, and disturbed sleep [11], with a global prevalence of 45–95% [12,13,14]. Heavy menstrual bleeding (HMB) affects up to 50% of adolescents and young people [6,15]. HMB can negatively affect daily activities and is often associated with embarrassment, and concerns about odour or leakage [16], as well as linked to iron deficiency following prolonged heavy bleeding [15]. Premenstrual syndrome including mood changes affects 20–75% globally, with high rates of mood disturbance, fatigue, reduced concentration and depressive symptoms prior to menstruation [6,17].

Menstrual-related symptoms are also known to affect sport and physical activity participation [18], with athletes sometimes missing training sessions and competitions due to pain or discomfort [19,20,21,22,23]. Supporting these findings, a large international study of more than 6800 physically active adult women of reproductive age found that the most commonly reported menstrual cycle symptoms were mood changes or anxiety, fatigue, stomach cramps, and breast pain or tenderness [23]. Greater symptom burden was also associated with an increased likelihood of missing or modifying training sessions and competitions. A study including both young and adult athletes found that menstrual complaints were more common among young athletes under the age of 18 years old [24]. A review study of adolescent girls found that menstruation can be a barrier to girls’ physical activity due to menstrual symptoms, lack of education and practical challenges such as clothing rules and access to period products [9]. Beyond physiological factors, participation and performance in sport may also be shaped by menstrual stigma and persistent taboos surrounding menstruation [9,25]. Menstrual stigma refers to socially embedded negative beliefs and attitudes toward menstruation and those who menstruate, rooted in prevailing cultural norms [26]. In this sense, menstrual stigma can be understood as a socially constructed framework in which menstruation is portrayed as unclean, embarrassing, and something that should be hidden from public view [26]. Several studies demonstrate that menstrual stigma constitute a barrier for communication about menstrual cycle between coaches and athletes [25,27]. Furthermore, studies involving adolescents indicate a need for enhanced support, specifically through educational initiatives targeting both athletes and coaches, as well as increased normalisation of menstruation within sporting contexts [18,22].

The majority of existing research on the menstrual cycle within sport contexts has predominantly focused on elite athletes and adult female athletes, particularly in relation to performance [28,29]. In studies including adolescents, the focus has predominantly been on high-performance or elite-level athletes [2]. Against this background, there is a clear need for research that explores menstrual health within organised sport from the perspectives of non-elite adolescent girls. In Sweden, floorball is one of the most practiced organised sports with more than 124,000 licenced athletes. Of these players, 37% are female, and among them approximately 58,500 are between 10 and 18 years old [30]. Floorball is a fast-paced indoor team sport played by two opposing teams, each with five field players and one goalkeeper on the court, using lightweight sticks and a hollow perforated plastic ball. The game involves frequent accelerations, sprinting, and rapid changes in direction. The present study aims to examine the impact of menstrual-related symptoms on sport participation and management strategies among adolescent girls playing floorball.

## 2. Materials and Method

### 2.1. Study Design and Data Collection

This cross-sectional study was conducted in Sweden between April and October 2025. Data were collected using an online survey for participants aged 15–18 years, and a paper survey for those aged 12–14 years, as parental consent was required for the younger group. The survey was distributed through floorball associations across five floorball districts in Sweden. The floorball associations, which consisted of team coaches and administrators, were asked to forward the questionnaire to their team members—girls aged 12 to 18 who play floorball. Participants aged 12–14 received a paper survey from their coach, which they brought home to obtain parental/guardian consent. Upon completion, the survey was returned to the research team at Karolinska Institutet by post using the prepaid envelope provided with the survey. Players aged 15–18 were invited to participate via a survey link using the REDCap, version 14.5.44 and 15.0.37, hosted at Karolinska Institutet [31,32]. Data were subsequently collected and managed using REDCap (Research Electronic Data Capture), which is a secure, web-based software platform designed to support data capture for research studies, providing (1) an intuitive interface for validated data capture; (2) audit trails for tracking data manipulation and export procedures; (3) automated export procedures for seamless data downloads to common statistical packages; and (4) procedures for data integration and interoperability with external sources. Because distribution occurred through floorball associations (coaches and administrators), the total number of eligible players who received the invitation is unknown, and a conventional response rate could therefore not be calculated.

### 2.2. Participants

**Figure 1:**
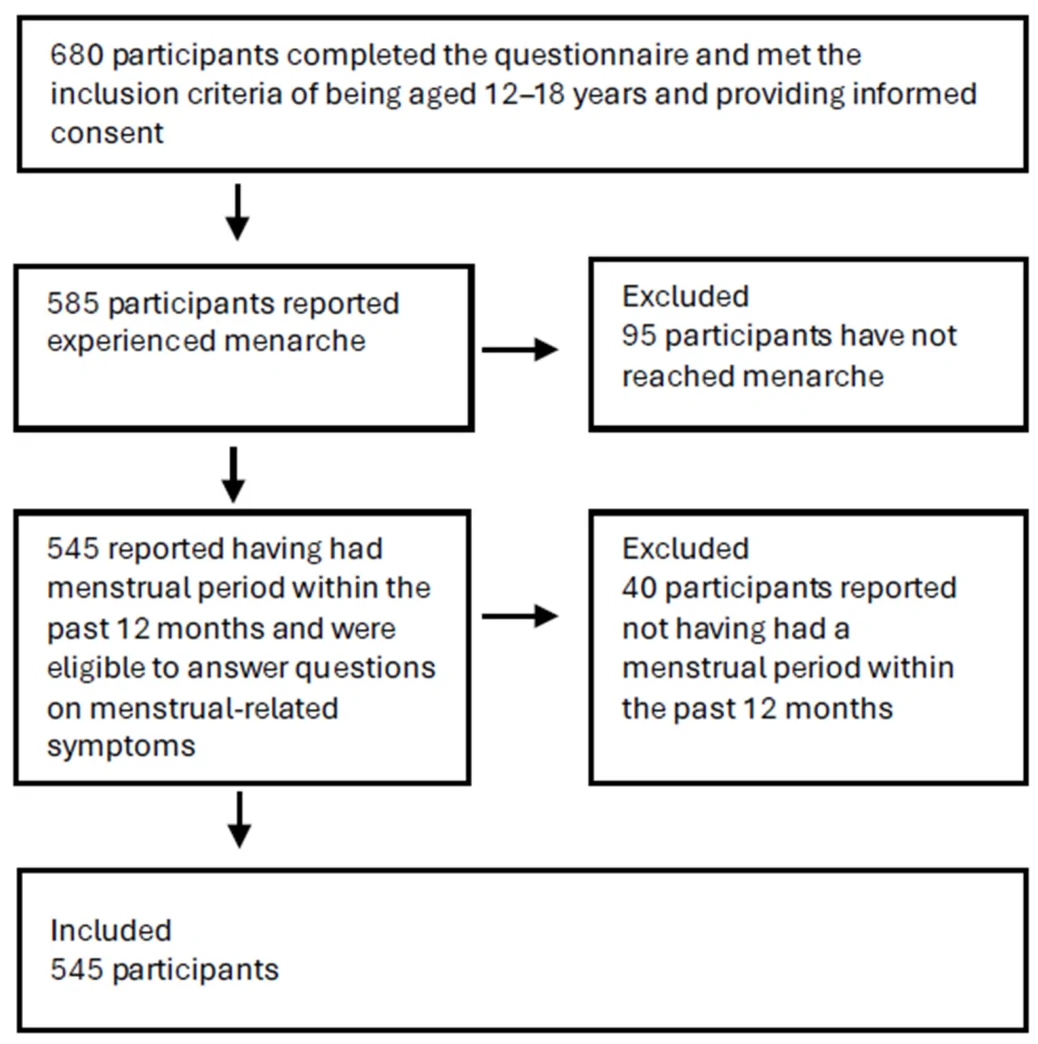
Flow chart of study participant selection.

A total of 680 adolescent female floorball players participated and completed the questionnaire. Of these, 86% reported having experienced menarche, while 14% had not yet reached menarche. Among those who had experienced menarche, 545 reported having had a menstrual period within the past 12 months and were included in the analyses, as they were eligible to answer questions on menstrual-related symptoms. Participants who reported no menstrual period in the past 12 months (*n* = 40) were instructed to skip questions on menstrual-related symptoms to minimise reporting bias and were therefore excluded from these analyses. Participants who had not yet reached menarche were also excluded. The majority of those who had not reached menarche were aged 12–14 years (93%). Figure 1 illustrates the participants selection criteria.

All participants were informed about the study aim and the procedures and provided informed consent. Written informed consent for the younger participants aged 12–14 years were provided by their parent/guardian and participants aged 15–18 years provided informed consent electronically before starting the questionnaire. The study was approved by the Swedish Ethical Review Authority (registration number: 2023-07178-02).

### 2.3. Questionnaire

The questionnaire contained 37 questions covering the following themes: socio-demographic issues, duration of playing floorball, self-rated health, menarche age, menstrual knowledge before menarche, menstrual-related symptoms, symptom management, performance in relation to menstruation, reasons for reduced performances and menstrual communication within the teams. Most of the items assessing menstrual-related symptoms were adapted from a previous study in Sweden conducted by the Swedish Public Health Agency [6]. A reference group consisting of two young floorball players and three coaches reviewed the survey and provided feedback to ensure its relevance to the floorball context. Subsequently, the questionnaire was piloted through cognitive interviews with five floorball players aged 12 to 18 years, followed by minor revisions based on their input. For example, we added a brief explanation of what contraceptives can be used for, as some participants were unfamiliar with the term.

Menstrual-related symptoms were assessed with the following question: During the past 6 months, how often have you experienced:

- menstrual pain
- heavy menstrual bleeding
- mood changes before or during menstruation
- ‘other’ menstrual complaints (e.g., headaches, tiredness, dizziness, concentration difficulties)?

The response options were as follows: Every or almost every menstruation/About half the times I have had menstruation/One or two few times the past 6 months/Never/Don’t know. The question did not focus on how long symptoms lasted during each menstruation, but on how many menstruations the various symptoms had occurred over the past six months.

Menstrual-related symptoms impact on floorball training/matches were assessed with the following question:

- During the past 6 months, have you experienced that your menstruation-related symptoms have affected your floorball training and matches? The response options were as follows: Every or almost every menstruation/About half the times I have had my menstruation/One or two times the past 6 months/Never/Don’t know.

Refraining from floorball training/matches was assessed with the following question:

- Have you ever refrained from training and matches due to menstrual-related symptoms. The question referred to both training sessions and matches, and these were not assessed separately. The response options were as follows: Yes/No/Don’t know.
- During the past 6 months, how often have you refrained from floorball training and matches due to menstrual-related symptoms? The response options were as follows: Two or more training sessions each month/One training session each month/One training session every other month/Occasional training session in the past six months/Don’t know. Other included questions are presented in detail in Supplementary S1 and Supplementary S2 provides an overview of the questionnaire topics.

### 2.4. Statistical Analyses

The data were analysed using IBM SPSS version 30. Descriptive analyses were conducted to report the frequencies and proportions of the characteristics and reported menstrual-related symptoms. Dependence between age groups (12–14 and 15–18 years) and all analysed variables were examined using the chi-square test, and differences in proportions were assessed using a z-test for proportions. Bivariate logistic regression was performed to calculate the crude odds ratios (OR), with 95% confidence intervals, to estimate the extent to which the explanatory variable (age group) affected outcome variables (menstrual-related symptoms, menstrual-related symptoms impact on participation and refraining from training/matches). Thereafter, multivariate logistic regression was used to adjust for the potential confounders (contraceptive use, age at menarche, menstrual pain). *p*-values less than 0.05 were considered significant.

In the initial regression models, birth control use was statistically significant with refraining from training/matches. This finding was contrary to our expectations, as we had anticipated that non-use of contraceptives would be associated with a higher likelihood of refraining from training due to menstrual-related symptoms.

As the underlying mechanism for this association was unclear, additional analyses were conducted based on the hypothesis that menstrual-related symptoms (bleeding, pain, mood changes, and other symptoms) could be associated with both birth control use and the outcome. Interaction terms were tested but excluded due to poor model robustness and limited interpretability. Associations between individual symptoms and birth control use were examined using chi-square tests, showing that only pain was significantly associated with birth control use. Pain was therefore included as a control variable in subsequent models. After adjustment for pain, birth control use remained statistically significant in some models, suggesting that the association could not be fully explained by pain alone. To assess robustness, alternative modelling approaches were applied, including binomial probit models and logistic regression methods accounting for separation. Several models showed issues with quasi-separation when pain was included, leading to unstable estimates. As this issue could not be adequately resolved, pain was excluded from certain models to ensure model stability.

## 3. Results

### 3.1. Sociodemographic Characteristics of Participants

The participants’ sociodemographic data are presented in Table 1. Most participants were aged 15–18 years (71.0%), while 29% were aged 12–14 years. The majority were born in Sweden with both parents also born in Sweden (86.9%). An additional 9.1% were born in Sweden with one parent born outside Sweden. Most participants (91.9%) had played floorball for four seasons (Supplementary S1, Table S1). Smaller proportions had played for three seasons (4.1%), and two seasons (2.8%), or were in their first season or had recently started (1.3%). Regarding weekly participation in floorball training and matches, half of the participants reported 4–6 h per week (50.2%). Approximately one third reported 7–9 h (28.3%), while 14.5% reported participating ≥10 h per week.

### 3.2. Menstrual Characteristics

The majority of participants (60.2%) reported menarche between ages 12 and 13. One quarter (25.1%) reported menarche at age 11 or younger and a smaller proportion (14.2%) reported onset at age 14 or older. Regular menstrual cycles (21–35 days) were reported among 60.8% of the participants, while 31.4% reported irregular cycles and 7.8% were unsure. Around one-quarter (27.6%) reported using contraceptives (Table 1). The most frequently reported reasons for contraceptive use were to stop menstruation (55.7%) and reduce menstrual pain (56.4%), followed by pregnancy prevention (45.0%), reduction in menstrual bleeding (36.4%), and management of mood swings or premenstrual symptoms (24.3%) (Supplementary S1, Table S3). The type of contraceptive methods is presented in Supplementary S1, Table S2.

### 3.3. Menstrual-Related Symptoms

Nearly 90% of participants reported experiencing menstrual-related symptoms at least one time during the past 6 months (Supplementary S1, Table S4). The most commonly occurring symptoms during every or approximately half of all menstruations during the past 6 months were menstrual pain and mood swings, reported by 72.2% and 75.3% of participants. Other menstrual-related symptoms (e.g., headache, fatigue, dizziness, concentration difficulties) were reported by 48% of participants, and 42.5% experienced heavy menstrual bleeding with similar frequency during the past 6 months. Table 2 presents the crude and adjusted logistic regression models examining the associations between age group, contraceptive use, age at menarche, and frequent menstrual-related symptoms. Crude analyses showed no significant associations between age group and frequent menstrual pain, heavy menstrual bleeding, mood swings or other menstrual-related symptoms. In the fully adjusted analyses, menarche at 11 years or younger (≤11 years) was associated with increased odds of several menstrual symptoms. Participants who had menarche at ≤11 years had higher odds of frequent menstrual pain compared with those who had menarche at 12 years or older (OR = 2.19, 95% CI 1.22–3.93; *p* = 0.008). Similarly, menarche at ≤11 years was significantly associated with frequent heavy menstrual bleeding (OR = 1.84, 95% CI 1.15–2.95; *p* = 0.010) and other menstrual-related symptoms such as headache, fatigue, dizziness, and concentration difficulties (OR = 2.06, 95% CI 1.24–3.40; *p* = 0.005). No significant associations were observed between age group, contraceptive use, and frequent menstrual-related symptoms. Similarly, no significant association was found between hours of training per week and the frequency of menstrual symptoms (Figure S1).

### 3.4. Impact of Menstrual-Related Symptoms on Training and Matches

**Figure 2:**
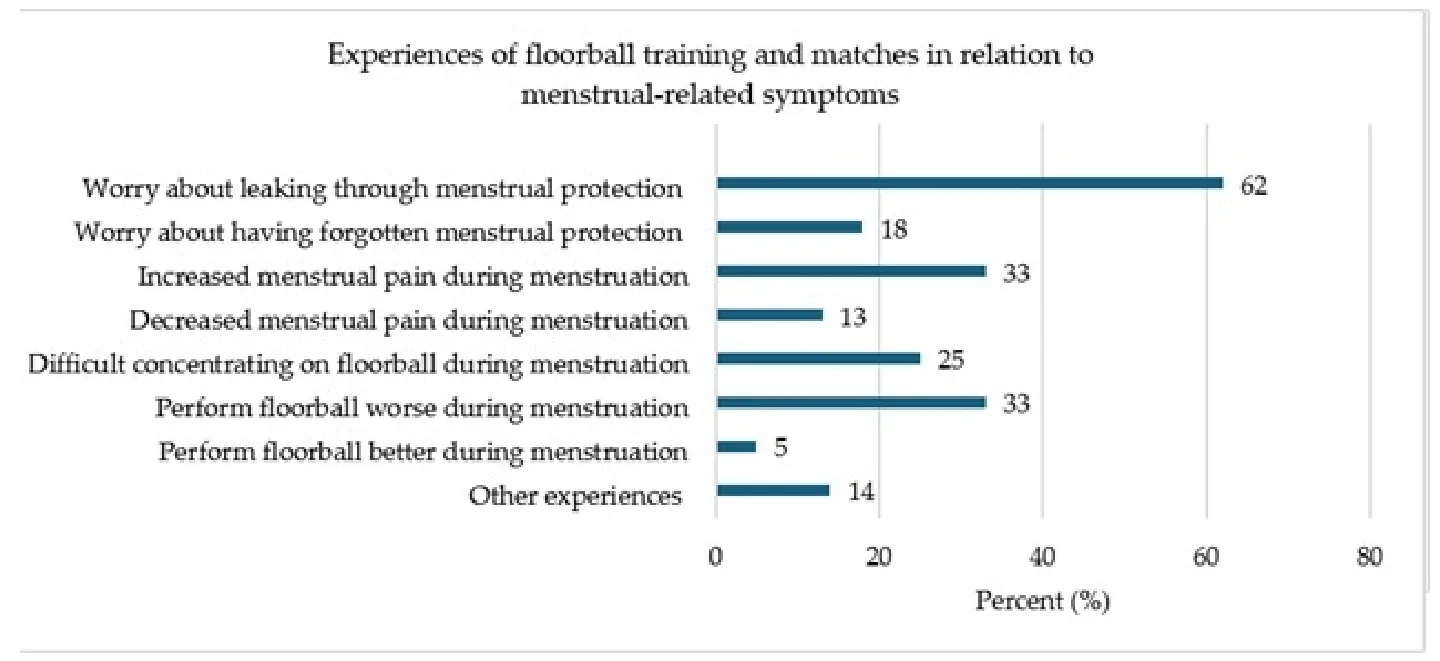
Reported experiences of floorball training and matches due to menstrual-related symptoms among all age group. Participants could report multiple reasons of experience.

Two-thirds of participants (62.2%) reported that menstrual-related symptoms affected their floorball training and matches during the past six months (Supplementary S1, Table S5). Figure 2 presents reported experiences of playing or competing in floorball during menstruation. Worry about leakage was the most frequently reported concern (62%). Difficulties concentrating during training and matches were reported by one-quarter of participants. Reported experiences of reduced performance (33%) during menstruation were more commonly reported than experiences of improved performance (5%). One-third of participants reported decreased menstrual pain, while 13% reported increased pain. No differences were observed between age groups, except that older participants (aged 15–18 years) more frequently reported concerns about forgetting menstrual products (*p* ≤ 0.01), and younger participants more frequently reported other menstrual-related experiences (*p* ≤ 0.05), (Supplementary S1, Table S6). In the adjusted model (Table 3), participants who reported frequent menstrual pain had eleven-fold higher odds of reporting an impact on training and matches (OR = 11.3, 95% CI 3.26–39.1; *p* ≤ 0.001).

One-third of the participants (n = 148) reported refraining (at least once) from training and matches due to menstrual-related symptoms (Supplementary S1, Table S7). Higher odds of refraining were observed among participants with menarche age 11 years or younger (OR = 1.67, 95% CI 1.00–2.78; *p* ≤ 0.05), those using contraceptives (OR = 3.52, 95% CI 2.08–5.97; *p* ≤ 0.001), and those reporting frequent menstrual pain (OR = 4.68, 95% CI 1.31–16.6; *p* ≤ 0.05). Of those who had ever refrained, 80% of them reported that they had done so during the past 6 months (Supplementary S1, Table S7). This included the following response options: a few times, and one session about every month or every other month. Around one-fourth refrained from one training session every month or one training session every other month.

**Figure 3:**
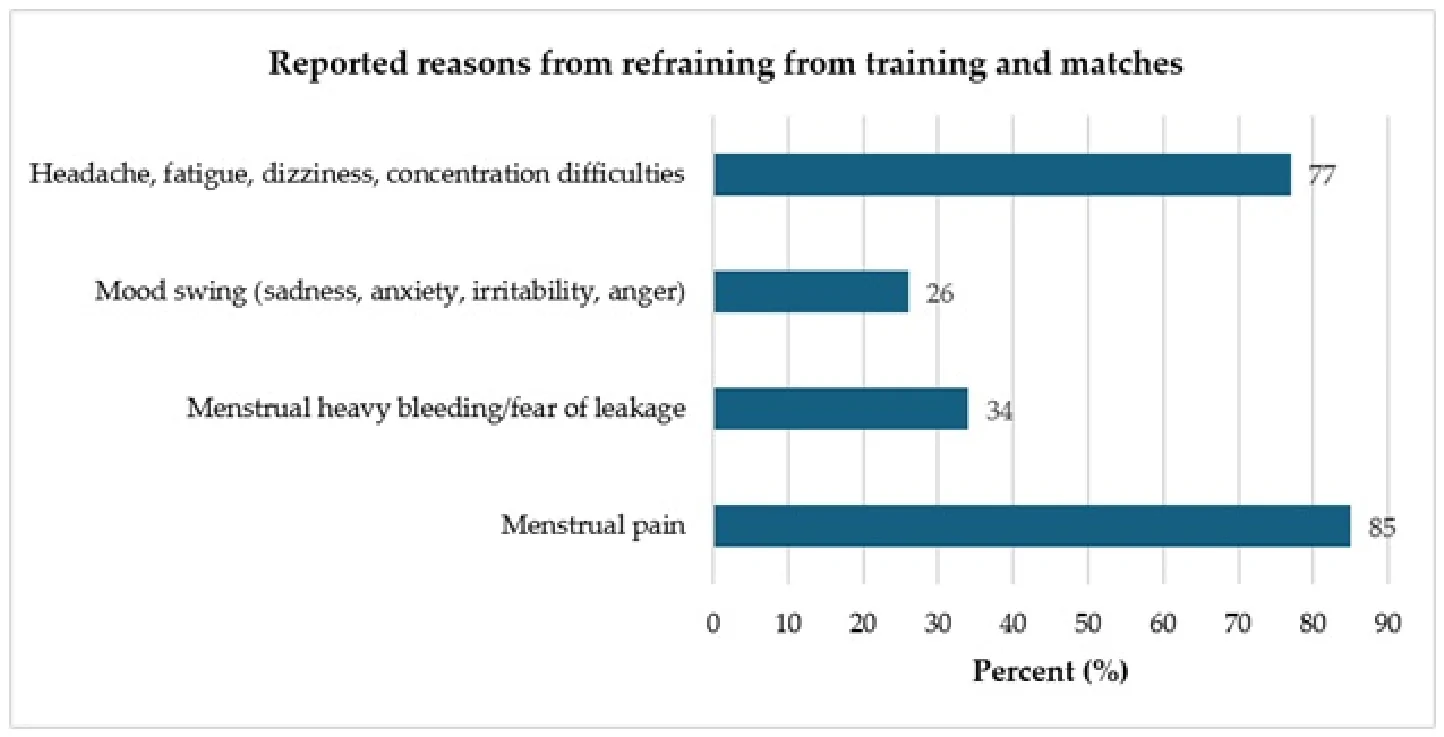
Reported reasons from refraining from training and matches among all age groups. Participants could report multiple reasons for refraining.

In the fully adjusted model, participants with menarche age at 11 years or younger (OR = 2.85, 95% CI 1.15–7.06; *p* ≤ 0.05) and participants aged 15–18 years old had higher odds (OR = 3.76, 95% CI 1.26–11.2; *p* ≤ 0.05) reporting frequent refraining (at least one training session every month). The most common reason for refraining was menstrual pain (78%), followed by headache, fatigue, dizziness and concentration difficulties (70.8%), heavy menstrual bleeding/fear of bleeding (31.1%) and mood swings (24.2%) (Figure 3). No differences were observed between age groups, except that older participants (aged 15–18 years) reported mood swings as a reason more frequently than the younger participants (aged 12–14 years) (*p* ≤ 0.05), (Supplementary S1, Table S8).

### 3.5. Healthcare Seeking, Perceived Adequacy of Care and Pain Management

The majority of the participants have not sought care for their menstrual-related symptoms (Supplementary S1, Table S9). One in five participants (20.4%) had sought healthcare for menstrual pain. Healthcare seeking for heavy menstrual bleeding was less common, with 11.4% reporting that they had consulted health services for this reason and 88.6% indicating they had not. Few (5%) had sought healthcare for mood swings related to menstruation. Across all menstrual-related symptoms, a higher proportion of participants aged 15–18 years reported having sought healthcare compared with those aged 12–14 years. Table S9 in Supplementary S1 presents the types of symptoms for which participants sought care, by age group.

Among participants who had sought care for menstrual pain (*n* = 105), 84.8% perceived that they received adequate care, while 9.5% felt they did not, and 5.7% were unsure. Similarly, among those who sought care for heavy menstrual bleeding (*n* = 59), 83.1% perceived the care as adequate, 10.2% as inadequate, and 6.8% were unsure. For mood-related symptoms *(n* = 25), 80.0% reported receiving adequate care, whereas 4.0% did not and 16.0% were unsure. Among participants seeking care for other menstrual-related symptoms (headache, fatigue, dizziness, concentration difficulties), 84.3% *(n* = 70) perceived the care as adequate, while 8.6% reported inadequate care, and 7.1% were unsure.

Use of pain-relief medication during the past six months was reported by 366 participants (Supplementary S1, Table S10). Of these, 40.1% used pain relief every for almost every period, and 17.7% used it during approximately every second period. Another 20.3% had used pain relief once or twice in the past six months.

### 3.6. Communication with Teammates and Coaches

Table S11 in Supplementary S1 presents data on participants’ communication about menstruation with teammates and coaches. A significantly higher proportion of the older group (73.5%) than the younger group (54.5%) reported having talked with teammates about menstruation (*p* ≤ 0.05). Communication with coaches was less common, with 76.9% of the younger group and 78.8% of the older group reporting that they had not spoken to their coach about menstruation. Perceived difficulty or embarrassment when talking about menstruation was significantly more common among 12–14-year-olds (31.7%) than among 15–18-year-olds (19.9%) (*p* ≤ 0.05). Most participants reported not having received information about menstruation from their coach or another adult within floorball, with a higher proportion in the older group (67.3%) than the younger group (54.7%) (*p* ≤ 0.05).

## 4. Discussion

This study is among the few studies that have examined the impact of menstrual-related symptoms on sport participation, experiences and management strategies among adolescent girls playing floorball. The findings of this study contribute to an improved understanding of how menstruation impacts girls’ participation and functioning in organised sport. We found that menstrual-related symptoms are highly prevalent among adolescent girls and impact their participation in floorball training and matches. Despite high symptom burden, the majority of participants have not sought care. Moreover, the findings suggest that girls with early onset of menarche at 11 years of age or younger reported higher odds of recurrent menstrual pain, heavy menstrual bleeding, and additional symptoms including headache, fatigue, dizziness, and difficulties with concentration. Girls who experienced early menarche had increased odds of refraining from floorball training and matches compared to those who experienced menarche later. These findings may indicate that girls with early menarche represent a subgroup that could require additional attention and support in youth sport.

Menstrual-related symptoms were highly prevalent among the majority of the participants in this study. The most common symptoms included menstrual pain, mood swings, and heavy menstrual bleeding, which aligns with previous studies in adolescent and adult athletes [33,34,35]. Our findings suggest that early menarche (≤11 years) was significantly associated with increased odds of recurrent menstrual pain, heavy menstrual bleeding, and other symptoms such as headache, fatigue, dizziness, and concentration difficulties. The observed associations are consistent with previous studies showing that early menarche is a risk factor for adolescent dysmenorrhea, with increased odds of frequent and severe menstrual pain [36,37,38,39]. However, to our knowledge, no previous studies in sport settings have specifically examined the association between age at menarche and menstrual-related symptom burden.

Another finding in our study was that early menarche was also associated with higher odds of frequent refraining (≥one session per month). Together, these findings suggest that girls with early menarche may experience greater challenges related to menstrual symptoms and sport participation, although future longitudinal studies are needed to better understand these relationships.

In line with our findings, previous studies in female athletes have shown that menstrual-related symptoms and disorders reduce training and competition performance, as well as attendance [2,22,33,40]. In our study, around two-thirds reported that their menstrual-related symptoms affected their training and matches. The most common reasons were worry about leaking and menstrual pain, which is consistent with previous research on elite athletes and as well as among non-elite athletes [18,22,28,41]. Worry about leakage reflects the persistent stigma surrounding menstrual blood [26], suggesting that sociocultural factors, alongside physical symptoms, continue to shape athletes’ experiences and participation in sport. However, no significant association was observed between weekly training hours and the frequency of menstrual symptoms.

Despite high symptom burden occurring monthly or almost, few have missed training and matches every month. However, one quarter reported missing training and matches at least once during the past half year, which is consistent with previous research [2,22], demonstrating that menstrual-related symptoms commonly disrupt sport participation among adolescent athletes. The most common reason for refraining was menstrual pain (85%) and headache, fatigue, dizziness and concentration difficulties (77%) among those who reported missed training and matches during the past half year. These findings are in line with previous studies among adolescents showing that menstrual pain is common and is associated with limited sport activity [42,43]. Moreover, the high prevalence of fatigue and dizziness among those who missed training and matches may also reflect the broader symptom burden associated with heavy menstrual bleeding, which has been linked to iron deficiency in adolescents [15,44].

Moreover, despite a high symptom burden and clear impact on sports participation, healthcare seeking for menstrual-related symptoms was low in the present study. To the best of our knowledge, there are no other studies on adolescent athletes’ healthcare-seeking in relation to menstrual symptoms. A study among adult athletes experiencing amenorrhea found that they do not seek care as the absence of menstruation was not perceived as a problem [45]. However, robust evidence from studies of girls and women in the general population worldwide demonstrates multiple barriers to healthcare-seeking. These include the normalisation of menstrual complaints [46,47,48,49,50,51], menstrual taboo and stigma [52], embarrassment and limited awareness of available treatment options [53] and lack of menstrual health knowledge, including uncertainty about what constitutes “normal” menstruation [54]. Furthermore, communication about menstruation with coaches was uncommon in this study, with more than three-quarters of the participants reporting that they had not discussed menstruation with their coach. This finding is consistent with previous research [2,27,55], demonstrating that menstruation remains a stigmatised and often avoided topic within youth sport [2,18]. A study of Swedish floorball coaches training girls aged 10–18 showed that menstruation was perceived as a sensitive and challenging topic to address, largely due to persistent stigma [56]. In this study, coaches wished to expand their knowledge about menstruation, particularly male coaches, who were perceived as needing greater understanding of the menstrual cycle.

In line with previous research [18,22,57], this study highlights the importance of menstrual health education within sport setting targeting both coaches and athletes, aimed at normalising discussions about menstrual health and fostering supportive environments in youth sport. Previous research has highlighted the need for improved education on the menstrual cycle, together with evidence-based management strategies to reduce disruptions and support performance [41]. Increasing awareness of the impact of the menstrual cycle on sports performance within sport settings may improve girls’ menstrual health literacy and better equip coaches and sporting organisations to support symptom management during training and matches. From a gender and equity perspective, these findings are particularly important, as menstrual stigma and silence may contribute to unequal sporting conditions for girls compared with boys. When menstrual health is not acknowledged or adequately supported within sporting environments, girls may be disadvantaged in their opportunities to participate, perform, and remain engaged in sport. Addressing menstrual health therefore extends beyond individual symptom management and should be understood as an issue of gender equity and inclusion within youth sport. Furthermore, considering the worldwide evidence demonstrating the menstrual stigma within sport settings [25], menstrual education efforts should include strategies on how coaches can initiate menstrual conversations and create an open environment where young athletes feel comfortable discussing their menstrual cycle. Such efforts may contribute to challenging gendered norms and reducing the silence surrounding menstruation in sport, ultimately supporting more inclusive and equitable sporting environments for adolescent girls.

### 4.1. Implications

The findings of this study have important implications for youth sport practice, menstrual health promotion, and coach education. Given the high prevalence of menstrual-related symptoms and their impact on participation, menstrual health should be recognised as a core component of athlete well-being in adolescent sport settings. Integrating menstrual health education into coach training programmes may help normalise discussions, reduce stigma, and facilitate supportive sport environments. Such educational efforts may support the early identification of girls with a high symptom burden and enable timely support to reduce avoidable missed training sessions and competitions. The low levels of communication with coaches highlight the need to normalise conversations about menstruation. In addition, menstrual health education for adolescent athletes may strengthen menstrual health knowledge and confidence, thereby reducing feelings of embarrassment and improving communication with coaches about the menstrual cycle. Nevertheless, menstrual health education in sports is essential for long-term health and gender equality. From a gender equity perspective, addressing menstrual health in sport is essential for creating fair and inclusive conditions for girls’ participation. When menstruation remains stigmatised or unsupported, girls risk being disadvantaged in their sporting experiences and opportunities compared with boys. By challenging taboos and integrating menstrual health into sport practice and education, adolescents can be empowered to better understand and manage their bodies, while coaches and sporting organisations can contribute to more equitable support systems and participation opportunities within youth sport.

### 4.2. Methodological Limitations

There are some limitations that need to be acknowledged in this study. A limitation of the study is that the survey was distributed through floorball associations, and the research team could not verify how many eligible players were reached. Therefore, no precise response rate could be calculated. This may have introduced selection bias, as players with greater interest in menstrual health, high burden of symptoms, or more supportive club environments may have been more likely to participate. Consequently, the prevalence of menstrual-related symptoms may have been overestimated if players experiencing such symptoms were more motivated to participate. This potential selection bias may have affected the external validity of the findings and should therefore be considered when interpreting the prevalence estimates and their generalizability. Furthermore, the study relied on self-reported data, which may be subject to recall and reporting bias. However, to minimise recall bias, participants were asked to report their experiences of menstrual symptoms over the past six months, and participants who reported no menstrual bleeding in the past 12 months were excluded from the analyses. The uneven age distribution, with a majority of participants aged 15–18 years (71%), may have influenced the observed associations, as age is likely related to both menstrual experiences and management strategies. In addition, some of the observed associations in Table 3, particularly those involving menstrual pain, early menarche and age group in relation to refraining from training and matches or perceived impact, were accompanied by relatively wide confidence intervals. These findings should therefore be interpreted with caution, as the wide intervals indicate limited precision of the estimates. Future studies with larger samples are needed to confirm these associations, Furthermore, participants were not asked whether they identified as transgender or non-binary, nor were data collected on self-reported experiences of disability—factors that may shape how menstrual symptoms are experienced in relation to sport participation.

## 5. Conclusions

This study demonstrates that menstrual-related symptoms are highly prevalent among adolescent girls playing floorball, which affect experiences of training and matches. A substantial proportion of participants reported recurrent symptoms, refraining from training sessions or matches, and low healthcare seeking despite high symptom burden. Early age at menarche was associated with increased symptom frequency and greater likelihood of refraining from training sessions or matches. These findings suggest that girls with early menarche may experience greater challenges related to menstrual symptoms and sport participation. Further research is needed to better understand these associations. Limited communication with coaches further highlights structural and social barriers to adequate support. Together, these findings emphasise the need for increased menstrual health awareness, early identification of girls at risk, and the development of supportive environments within organised youth sport. Integrating menstrual health into sport settings targeting both coaches and adolescent is essential to support adolescent athletes and promote gender equality in sport.

## Figures and Tables

**Table 1:** Sociodemographic background among participants, frequencies and proportions.

Variable All Participants (*n* = 545)	*n*	(%)
**Age**		
12–14 years	158	(29.0)
15–18 years	387	(71.0)
**Country of birth**		
Born in Sweden with both parents born in Sweden	470	(86.9)
Born in Sweden with one parent born outside Sweden	49	(9.1)
Born in Sweden with both parents born outside Sweden	13	(2.4)
Born outside Sweden	9	(1.7)
**Age at menarche** Median menarche age = 12		
≤11	131	(25.1)
12–13	314	(60.2)
≥14	74	(14.2)
Don’t know	<5	
**Regular menstruation during the past 12 months (21–35-day cycle)**		
Regular menstruation	329	(60.8)
Irregular menstruation	170	(31.4)
Don’t know	42	(7.8)
**Contraceptive use during the past 6 month**		
Yes	131	(27.6)
No	338	(71.3)
Don’t know	<5	

**Table 2:** Factors associated with frequent menstrual-related symptoms. Crude and fully adjusted model.

Type of Menstrual Symptom	*n*		Crude Models	*n*		Adjusted Models
**Frequent menstrual** **pain**			**OR (95%CI)**			**OR (95%CI)**
		**Age group**			**Age group**	
	147	12–14	1	139	12–14	1
	350	15–18	0.79 (0.50–1.25)	284	15–18	0.90 (0.53–1.52)
					**Contraceptive use**	
				305	No	1
				118	Yes	1.12 (0.66–1.91)
					**Menarche age**	
				313	≥12	1
				110	≤11	2.19 (1.22–3.93) **
**Frequent menstrual bleeding**		**Age group**			**Age group**	
	135	12–14	1	130	12–14	1
	319	15–18	1.35 (0.90–2.02)	263	15–18	1.54 (0.96–2.48)
					**Contraceptive use**	
				284	No	1
				109	Yes	1.01 (0.62–1.63)
					**Menarche age**	
				289	≥12	1
				104	≤11	1.84 (1.15–2.95) *
**Frequent mood swings before or during menstruation**		**Age group**			**Age group**	
	134	12–14	1	126	12–14	1
	320	15–18	1.52 (0.87–2.65)	258	15–18	1.60 (0.82–3.13)
					**Contraceptive use**	
				275	No	1
				109	Yes	0.94 (0.45–1.94)
					**Menarche age**	
				262	≥12	1
				102	≤11	1.31 (0.66–2.61)
**Other frequent symptoms before and during menstruation (e.g., headache, fatigue, dizziness, concentration difficulties)**		**Age group**			**Age group**	
	142	12–14	1	133	12–14 years	1
	280	15–18	0.80 (0.53–1.21)	231	15–18 years	0.90 (0.56–1.46)
					**Contraceptive use**	
				268	No	1
				96	Yes	1.18 (0.70–1.99)
					**Menarche age**	
				262	≥12	1
				102	≤11	2.06 (1.24–3.40) **

1 = Frequent symptoms, defined as occurring in every, almost every, or about half of all menstruations during the past 6 months. OR = odds ratio, * *p*-value ≤ 0.05, ** *p*-value ≤ 0.01.

**Table 3:** Factors associated with perceived impact and participation of menstrual-related symptoms on floorball training and matches. Crude and fully adjusted model.

	*n*		Crude Models	*n*		Adjusted Models
**Perceived impact of menstrual-related symptoms on floorball training and matches in the 6 past months**			**OR (95%CI)**			**OR (95%CI)**
		**Age group**			**Age group**	
	147	12–14	1	131	12–14	1
	308	15–18	1.51 (1.00–2.28) *	258	15–18	1.36 (0.81–2.30)
					**Contraceptive use**	
				282	No	1
				107	Yes	1.42 (0.84–2.42)
					**Menarche age**	
				288	≥12	1
				101	≤11	0.96 (0.57–1.60)
					**Menstrual pain**	
				27	Never	1
				76	1–2 times in the past six months	1.77 (0.46–6.80)
				88	About half of all periods in the past six months	3.85 (1.05–14.0) *
				198	Every or almost every period	11.2 (3.26–39.1) ***
**Refrained (at least once) from floorball training and matches due to menstrual-related symptoms**		**Age group**			**Age group**	
	153	12–14	1	134	12–14	1
	332	15–18	1.36 (0.88–2.08)	277	15–18	0.95 (0.54–1.66)
					**Contraceptive use**	
				296	No	1
				115	Yes	3.52 (2.08–5.97) ***
					**Menarche age**	
				302	≥12	1
				109	≤11	1.67 (1.00–2.78) *
					**Menstrual pain**	
				26	Never	1
				78	1–2 times in the past six months	1.68 (0.42–6.67)
				94	About half of all periods in the past six months	2.87 (0.75–10.9)
				213	Every or almost every period	4.68 (1.31–16.6) *
**Refrained frequently ^1^ from training and matches due to menstrual-related symptoms in the past 6 months ^2^**		**Age group**			**Age group**	
	60	12–14	1	54	12–14	1
	104	15–18	2.51 (1.06–5.93) *	84	15–18	3.76 (1.26–11.2) *
					**Contraceptive use**	
				83	No	1
				55	Yes	0.65 (0.25–1.69)
					**Menarche age**	
				95	≥12	1
				43	≤11	2.85 (1.15–7.06) *

^1^ Refraining frequently defined as every month during the past 6 months. ^2^ Menstrual pain was excluded from the model due to quasi-complete separation, which resulted in unstable parameter estimates. OR = odds ratio, * *p*-value ≤0.05, ***p-value ≤ 0.001.

## Data Availability

The dataset presented in this article are not readily available because they may contain information that could compromise the privacy of study participant. Requests to access the datasets should be directed to eva.akerman.2@ki.se.

## Supplementary Materials

The following are available online at https://www.mdpi.com/article/10.3390/sports14080358/s1, Supplementary S1 (Figure S1: Correlation between training and matches exposure (hours/week) and frequent menstrual symptoms. Frequent symptoms, defined as occurring in every, almost every, or about half of all menstruations during the past 6 months, Table S1: Years and weakly hours of playing floorball, Table S2: Type of contraceptive methods usage, Table S3: Reasons for using contraception ^1^, Table S4: Menstrual-related symptoms during the past 6 months by age group, Table S5: Experiences of training affected by menstruation-related symptoms by age group, Table S6: Which of the following have you experienced during floorball training and matches? Multiple responses could be selected, Table S7: Rrefraining from floorball training and matches due to menstruation-related symptoms by age group, Table S8: Reasons for refraining from floorball training and matches during the past months. Multiple responses could be selected, Table S9: Sought care for menstruation-related symptoms by age group and total, Table S10: Over the past 6 months, how often have you taken pain-relieving medication for menstrual pain? For example, paracetamol (Alvedon), ibuprofen (Ipren), or naproxen, Table S11: Communication with teammates and coaches by age group); Supplementary S2. Description of questionnaire topics.
