# Supplementary material for: Menstrual-Related Symptoms, Early Menarche, and Their Impact on Sport Participation in Adolescent Floorball Players: A Cross-Sectional Study in Sweden
Source: Sports (Basel). 2026 Aug 18;14(8):358. doi: 10.3390/sports14080358 (PMC13517096; doi:10.3390/sports14080358)

# Supplementary 1



Title: Menstrual-Related Symptoms, Early Menarche, and Their Impact on Sport Participation in Adolescent Floorball Players: A Cross-Sectional Study in Sweden

**Table S1. Years and weakly hours of playing floorball.**

| | *n* | (%) |
|---|---|---|
| **Years of experiences in floorball** | | |
| 1 season or has recently started | 7 | (1.3) |
| 2 seasons | 15 | (2.8) |
| 3 seasons | 22 | (4.1) |
| 4 seasons | 498 | (91.9) |
| | | |
| **Weekly hours of floorball training and matches** | | |
| 1-3 hours | 38 | (7.0) |
| 4-6 hours | 271 | (50.2) |
| 7-9 hours | 153 | (28.3) |
| ≥ 10 hours | 78 | (14.5) |

**Table S2. Type of contraceptive methods usage.**

| | *n* | (%) |
|---|---|---|
| Use of oral contraceptives (combined pill or progestin-only pill) | 115 | (82.1) |
| Use of contraceptive implant | 17 | (12.1) |
| Use of hormonal intrauterine device | 8 | (5.7) |
| Use of other contraceptive methods | 7 | (5.0) |
| Use of vaginal ring | >5 | |
| Don't know | >5 | |

**Table S3. Reasons for using contraception[1].**

| | *n* | (%) |
|---|---|---|
| To stop menstruation | 78 | (55.7) |
| To reduce menstrual bleeding | 51 | (36.4) |
| To reduce menstrual pain | 79 | (56.4) |
| To reduce mood swing or PMS | 34 | (24.3) |
| Pregnancy prevention | 63 | (45.0) |
| Other reasons | 9 | (6.4) |
| Don't know | >5 | |

[1] Participants could report multiple reasons for using contraceptive

Title: Menstrual-Related Symptoms, Early Menarche, and Their Impact on Sport Participation in Adolescent Floorball Players: A Cross-Sectional Study in Sweden

**Table S4. Menstrual-related symptoms during the past 6 months by age group.**

| | **Total** | | **12–14 years** | | **15–18 years** | |
|---|---|---|---|---|---|---|
| **Frequency of menstrual pain** | ***n* 518** | **(%)** | ***n* 154** | **(%)** | ***n* 364** | **(%)** |
| Every or almost every period | 266 | (51.4) | 74 | (48.1) | 192 | (52.7) |
| About every other time I've had my period | 108 | (20.8) | 41 | (26.6)* | 67 | (18.4)* |
| 1-2 times in the past six month | 90 | (17.4) | 27 | (17.5) | 63 | (17.3) |
| Never | 33 | (6.4) | >5 | | 28 | (7.7) |
| Don't know | 21 | (4.1) | 7 | (4.5) | 14 | (3.8) |

| **Frequency of heavy menstrual bleeding** | **Total** | | **12–14 years** | | **15–18 years** | |
|---|---|---|---|---|---|---|
| | ***n* 516** | **(%)** | ***n* 151** | **(%)** | ***n* 365** | **(%)** |
| Every or almost every period | 120 | (23.3) | 28 | (18.5) | 92 | (25.2) |
| About every other time I've had my period | 99 | (19.2) | 30 | (19.9) | 69 | (18.9) |
| 1-2 times in the past six month | 111 | (21.5) | 27 | (17.9) | 84 | (23.0) |
| Never | 124 | (24.0) | 50 | (33.1)** | 74 | (20.3)** |
| Don't know | 62 | (12.0) | 16 | (10.6) | 46 | (12.6) |

| **Frequency of mood swings** | **Total** | | **12–14 years** | | **15–18 years** | |
|---|---|---|---|---|---|---|
| | ***n* 518** | **(%)** | ***n* 154** | **(%)** | ***n* 364** | **(%)** |
| Every or almost every period | 298 | (57.5) | 91 | (59.1) | 207 | (56.9) |
| About every other time I've had my period | 92 | (17.8) | 19 | (12.3)* | 73 | (20.1)* |
| 1-2 times in the past six month | 34 | (6.6) | 14 | (9.1) | 20 | (5.5) |
| Never | 30 | (5.8) | 10 | (6.5) | 20 | (5.5) |
| Don't know | 64 | (12.4) | 20 | (13.0) | 44 | (12.1) |

| **Frequency of other symptoms e.g. headache, fatigue, dizziness, concentration difficulties** | ***n* 521** | **(%)** | ***n* 157** | **(%)** | ***n* 364** | **(%)** |
|---|---|---|---|---|---|---|
| Every or almost every period | 158 | (30.3) | 43 | (27.4) | 115 | (31.6) |
| About every other time I've had my period | 92 | (17.7) | 46 | (29.3)* | 46 | (12.6)* |
| 1-2 times in the past six month | 63 | (12.1) | 15 | (9.6) | 48 | (13.2) |
| Never | 109 | (20.9) | 38 | (24.2) | 71 | (19.5) |
| Don't know | 99 | (19.0) | 15 | (9.6)*** | 84 | (23.1)*** |

1 Differences in proportions between groups were assessed using a z-test for proportions, with statistical significance set at $p \leq 0.05$.

*= $p \leq 0.05$, **= $p \leq 0.01$, ***= $p \leq 0.001$

Title: Menstrual-Related Symptoms, Early Menarche, and Their Impact on Sport Participation in Adolescent Floorball Players: A Cross-Sectional Study in Sweden

**Table S5. Experiences of training affected by menstruation-related symptoms by age group.**

| | Total | | 12–14 years | | 15–18 years | |
|---|---|---|---|---|---|---|
| | ***n* 503** | **(%)** | ***n* 157** | **(%)** | ***n* 346** | **(%)** |
| **Have you experienced that your menstruation-related symptoms have affected your floorball training and matches in the past 6 months?** | | | | | | |
| Every or almost every period | 69 | (13.7) | 19 | (12.1) | 50 | (14.5) |
| About every other month I've had my period | 110 | (21.9) | 29 | (18.5) | 81 | (23.4) |
| 1-2 times in the past six month | 134 | (26.6) | 45 | (28.7) | 89 | (25.7) |
| Never | 141 | (28.0) | 53 | (33.8) | 88 | (25.4) |
| Don't know | 49 | (9.7) | 11 | (7.0) | 38 | (11.0) |

Differences in proportions between groups were assessed using a z-test for proportions, with statistical significance set at p ≤ 0.05.
*= p ≤ 0.05, **= p ≤ 0.01, ***= p ≤ 0.001

**Table S6. Which of the following have you experienced during floorball training and matches?**
Multiple responses could be selected.

| | Total | | 12-14 years | | 15-18 years | |
|---|---|---|---|---|---|---|
| | ***n*** | | ***n*** | **(%)** | ***n*** | **(%)** |
| I worry about leaking through my menstrual protection | 336 | (61.7) | 96 | (60.8) | 240 | (62.0) |
| I worry about having forgotten menstrual protection | 99 | (18.2) | 18 | (11.4)** | 81 | (20.9)** |
| I perform floorball better when I have my period | 27 | (5.0) | 8 | (5.1) | 19 | (4.9) |
| I perform floorball worse when I have my period | 179 | (32.8) | 56 | (35.4) | 123 | (31.8) |
| I have difficulty concentrating on floorball when I have my period menstruation | 134 | (24.6) | 37 | (23.4) | 97 | (25.1) |
| I experience more menstrual pain when I play floorball during my period | 70 | (12.8) | 20 | (12.7) | 50 | (12.9) |
| I experience less menstrual pain when I play floorball during my period | 180 | (33.0) | 61 | (38.6) | 119 | (30.7) |
| Other experiences | 74 | (13.6) | 30 | (19.0)* | 44 | (11.4)* |

Differences in proportions between groups were assessed using a z-test for proportions, with statistical significance set at p ≤ 0.05.
*= p ≤ 0.05, **= p ≤ 0.01, ***= p ≤ 0.001

Title: Menstrual-Related Symptoms, Early Menarche, and Their Impact on Sport Participation in Adolescent Floorball Players: A Cross-Sectional Study in Sweden

**Table S7. Rrefraining from floorball training and matches due to menstruation-related symptoms by age group.**

| | **Total** | | **12–14 years** | | **15–18 years** | |
|---|---|---|---|---|---|---|
| | ***n* 504** | **(%)** | ***n* 157** | **(%)** | ***n* 346** | **(%)** |
| **Have you ever refrained from floorball training and matches due to menstruation-related problems?** | | | | | | |
| Yes | 148 | (29.4) | 40 | (25.3) | 108 | (31.2) |
| No | 337 | (66.9) | 113 | (71.5) | 224 | (64.7) |
| Don't know | 19 | (3.8) | >5 | (3.2) | 14 | (4.0) |
| **How often have you refrained from floorball training and matches due to menstruation-related problems during the past 6 months?** | | | | | | |
| 2 or more times every month | 0 | | 0 | | 0 | |
| One training session every month | 18 | (10.7) | 5 | (8.3) | 13 | (12.0) |
| One training session every other month | 19 | (11.3) | 3 | (5.0) | 16 | (14.8) |
| A few training sessions in the past six months | 97 | (57.7) | 36 | (60.0) | 61 | (56.5) |
| Never | 30 | (17.9) | 16 | (26.7)* | 14 | (13.0)* |
| Don't know | 4 | (2.4) | 0 | | 4 | (3.7) |

Differences in proportions between groups were assessed using a z-test for proportions, with statistical significance set at p ≤ 0.05.
*= p ≤ 0.05, **= p ≤ 0.01, ***= p ≤ 0.001

**Table S8. Reasons for refraining from floorball training and matches during the past months.**
Multiple responses could be selected.

| | **Total** | | **12–14 years** | | **15–18 years** | |
|---|---|---|---|---|---|---|
| | ***n*** | **(%)** | ***n*** | **(%)** | ***n*** | **(%)** |
| Refrained due to menstrual pain | 126 | (78.3) | 32 | (80.0) | 94 | (77.7) |
| Refrained due to heavy bleeding or fear of bleeding through | 50 | (31.1) | 11 | (27.5) | 39 | (32.2) |
| Refrained due to mood swings (sadness, anxiety, irritability, anger) | 39 | (24.2) | 15 | (37.5)* | 24 | (19.8)* |
| Refrained due to other symptoms e.g. headache, fatigue, dizziness, concentration difficulties | 114 | (70.8) | 33 | (82.5) | 81 | (66.9) |

Differences in proportions between groups were assessed using a z-test for proportions, with statistical significance set at p ≤ 0.05.
*= p ≤ 0.05, **= p ≤ 0.01, ***= p ≤ 0.001

Title: Menstrual-Related Symptoms, Early Menarche, and Their Impact on Sport Participation in Adolescent Floorball Players: A Cross-Sectional Study in Sweden

**Table S9. Sought care for menstruation-related symptoms by age group and total.**

| | **Total** | | **12–14 years old** | | **15–18 years old** | |
|---|---|---|---|---|---|---|
| | ***n*** | **(%)** | ***n*** | **(%)** | ***n*** | **(%)** |
| **Have you ever sought care for menstrual pain?** | | | | | | |
| Yes | 106 | (20.4) | 11 | (7.1)*** | 95 | (26.1)*** |
| No | 413 | (79.6) | 144 | (92.9)*** | 269 | (73.9)*** |
| **Did you receive the support you needed?** | | | | | | |
| Yes | 96 | (76.2) | 15 | (46.9)*** | 81 | (86.2)*** |
| No | 16 | (12.7) | 6 | (18.8) | 10 | (10.6) |
| Don't know | 14 | (11.1) | 11 | (34.4)*** | < 5 | |
| **Have you ever sought care for heavy menstrual bleeding?** | | | | | | |
| Yes | 59 | (11.4) | 10 | (6.3)* | 49 | (13.6)* |
| No | 460 | (88.6) | 148 | (93.7)* | 312 | (86.4)* |
| **Did you receive the support you needed?** | | | | | | |
| Yes | 50 | (66.7) | 9 | (34.6)*** | 41 | (83.7)*** |
| No | 12 | (16.0) | 7 | (26.9) | < 5 | |
| Don't know | 13 | (17.3) | 10 | (38.5)*** | < 5 | |
| **Have you ever sought care for mood swings?** | | | | | | |
| Yes | 26 | (5.0) | < 5 | | 25 | (6.9)** |
| No | 493 | (95) | 155 | (99.4)** | 338 | (93.1)** |
| **Did you receive the support you needed?** | | | | | | |
| Yes | 21 | (50.0) | < 5 | | 19 | (79.2)*** |
| No | 8 | (19.0) | 7 | (38.9)** | < 5 | |
| Don't know | 13 | (31.0) | 9 | (50.0)* | < 5 | |
| **Have you ever sought care for other menstrual related symptoms[1]?** | | | | | | |
| Yes | 70 | (13.4) | 8 | (5.1)*** | 62 | (17.0)*** |
| No | 451 | (86.6) | 149 | (94.9)*** | 302 | (83.0)*** |
| **Did you receive the support you needed?** | | | | | | |
| Yes | 61 | (70.1) | 10 | (40.0)*** | 51 | 82.3)*** |
| No | 13 | (14.9) | 7 | (28.0)* | 6 | (9.7)* |
| Don't know | 13 | (14.9) | 8 | (32.0)** | < 5 | |

[1] E.g. headache, fatigue, dizziness, concentration difficulties
Differences in proportions between groups were assessed using a z-test for proportions, with statistical significance set at $p \leq 0.05$.
*= $p \leq 0.05$, **= $p \leq 0.01$, ***= $p \leq 0.001$

Title: Menstrual-Related Symptoms, Early Menarche, and Their Impact on Sport Participation in Adolescent Floorball Players: A Cross-Sectional Study in Sweden

**Table S10. Over the past 6 months, how often have you taken pain-relieving medication for menstrual pain?** For example, paracetamol (Alvedon), ibuprofen (Ipren), or naproxen.

| | Total | | 12-14 years | | 15-18 years | |
|---|---|---|---|---|---|---|
| | ***n*** | **(%)** | ***n*** | **(%)** | ***n*** | **(%)** |
| Every or almost every period | 189 | (39.9) | 46 | (30.1)** | 143 | (44.5)** |
| About every other month I've had my period | 83 | (17.5) | 27 | (17.6) | 56 | (17.4) |
| 1-2 times in the past six month | 95 | (20.0) | 23 | (15.0) | 72 | (22.4) |
| Never | 92 | (19.4) | 49 | (32.0)** | 43 | (13.4)** |
| Don't know | 15 | (3.2) | 8 | (5.2) | 7 | (2.2) |

Differences in proportions between groups were assessed using a z-test for proportions, with statistical significance set at $p \leq 0.05$. *= $p \leq 0.05$, **= $p \leq 0.01$, ***= $p \leq 0.001$

**Table S11. Communication with teammates and coaches by age group.**

| | **Total** *n* (%) | **Age 12-14 years** *n* (%) | **Age 15-18 years** *n* (%) |
|---|---|---|---|
| **Have talked with teammate about menstruation** | | | |
| Yes | 324 (67.6) | 84 (54.5)*** | 250 (73.5)*** |
| No | 119 (24.1) | 53 (34.4) *** | 66 (19.4) *** |
| Don't know | 17 (8.3) | 17 (11.0) | 24 (7.1) |
| **Have talked with coach about menstruation** | | | |
| Yes | 87 (17.5) | 31 (19.9) | 56 (16.5) |
| No | 388 (78.2) | 120 (76.9) | 268 (78.8) |
| Don't know | 21 (4.2) | < 5 | 16 (4.7) |
| **Perceived difficulty or embarrassment talking about menstruation** | | | |
| Yes | 113 (23.5) | 46 (31.7)** | 67 (19.9)** |
| No | 273 (56.8) | 70 (48.3)* | 203 (60.4)* |
| Don't know | 95 (19.8) | 29 (20.0) | 66 (19.6) |
| **Have received information about menstruation from coach or adult within the floorball** | | | |
| Yes | 125 (25.8) | 50 (33.8)** | 75 (22.3)** |
| No | 307 (63.4) | 81 (54.7)** | 226 (67.3)** |
| Don't know | 52 (10.7) | 17 (11.5) | 35 (10.4) |

Differences in proportions between groups were assessed using a z-test for proportions, with statistical significance set at $p \leq 0.05$.
*= $p \leq 0.05$, **= $p \leq 0.01$, ***= $p \leq 0.001$

Title: Menstrual-Related Symptoms, Early Menarche, and Their Impact on Sport Participation in Adolescent Floorball Players: A Cross-Sectional Study in Sweden

**Figure S1. Correlation between training and matches exposure (hours/week) and frequent menstrual symptoms.** Frequent symptoms, defined as occurring in every, almost every, or about half of all menstruations during the past 6 months.

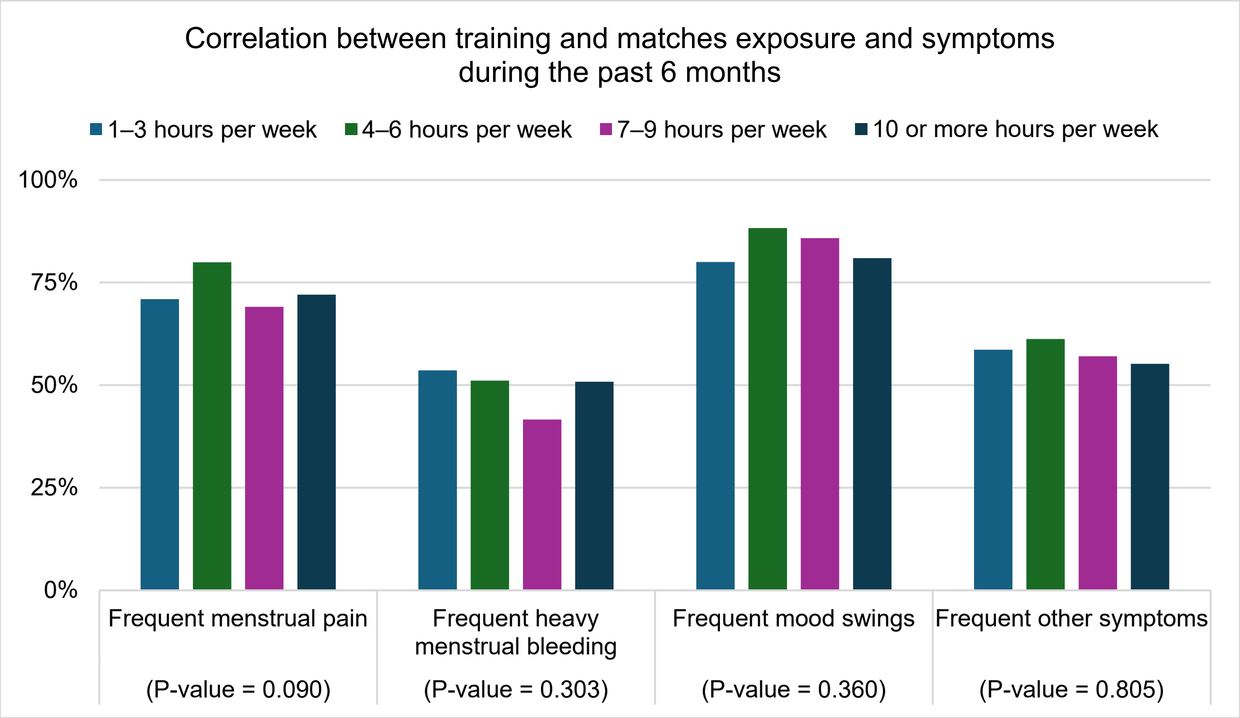

Supplement: Supplementary file 1 [file sports-14-00358-s001.zip › sports-4398237-supplementary/Åkerman et al 2026 Supplementary S1.pdf]
