# Supplementary material for: Menstrual-Related Symptoms, Early Menarche, and Their Impact on Sport Participation in Adolescent Floorball Players: A Cross-Sectional Study in Sweden
Source: Sports (Basel). 2026 Aug 18;14(8):358. doi: 10.3390/sports14080358 (PMC13517096; doi:10.3390/sports14080358)
Supplement: Supplementary file 1 [file sports-14-00358-s001.zip › sports-4398237-supplementary/Åkerman et al 2026 Supplementary S2.pdf]

Title: Menstrual-Related Symptoms, Early Menarche, and Their Impact on Sport Participation in Adolescent Floorball Players: A Cross-Sectional Study in Sweden

# Supplementary 2. Description of questionnaire topics

This supplementary material provides an English description of the topics covered in the original Swedish questionnaire. Researchers wishing to use the questionnaire may contact the corresponding author for further information eva.akerman.2@ki.se

**Participant background and floorball participation:** Questions cover age, floorball district, country of birth of the participant and parent(s), duration of floorball participation, and the amount of time spent in training.

**General health and menstrual history:** Questions assess self-rated health, menarche status and age at menarche, knowledge and preparedness before menarche, experiences of the first menstrual period, menstrual regularity, and menstruation during the past 12 months.

**Menstrual-related symptoms and healthcare-seeking:** Questions cover the frequency of menstrual pain, heavy menstrual bleeding, mood swings, and other menstrual-related symptoms. Additional questions assess the use of pain-relieving medication, healthcare seeking for menstrual concerns, and whether participants received the support or care they needed.

**Menstruation and floorball participation:** Questions examine the impact of menstrual-related symptoms on floorball training and matches, perceived effects of menstruation on performance and concentration, concerns related to menstrual management during sports participation, menstrual-related absence from training or competition, reasons for non-participation, and the influence of menstruation on other leisure activities.

**Support within floorball:** Questions assess communication about menstruation with teammates and coaches, perceived support from coaches, and the availability of menstrual symptom-related training adaptations within the floorball environment.

**Information and communication about menstruation:** Questions explore attitudes towards the provision of menstrual products, pain-relief medication, and menstrual health information within floorball clubs. Additional questions assess previous communication about menstruation in the sports setting, comfort discussing menstruation, information needs related to menstruation and training, and preferred sources of menstrual health information.

**Contraception:** Questions assess contraceptive use, types of contraceptive methods used, and reasons for use, including menstrual symptom management and pregnancy prevention.
